# Long noncoding RNA X‐inactive specific transcript (lncRNA XIST) inhibits hepatic insulin resistance by competitively binding microRNA‐182‐5p

**DOI:** 10.1002/iid3.969

**Published:** 2023-11-07

**Authors:** Guoqing Zhong, Qingping Yang, Yihua Wang, Yuan Liang, Xiaojing Wang, Dongli Zhao

**Affiliations:** ^1^ Hepatology Department First People's Hospital Nanyang China; ^2^ Endocrinology Department First People's Hospital Nanyang China

**Keywords:** lncRNA X‐inactive specific transcript, insulin‐like growth factor‐1 receptor, insulin resistance, miR‐182‐5p

## Abstract

**Background:**

What is highlighted in this study refers to the role and molecular mechanism of long noncoding RNA (lncRNA) X‐inactive specific transcript (XIST) in cells with insulin resistance (IR).

**Methods:**

In this study, LX‐2 cells were applied to establish IR model in vitro. The expressions of lncRNA XIST, phosphoenolpyruvate carboxykinase (PEPCK,) and glucose‐6‐phosphatase (G6Pase) were quantified by quantitative reverse transcription polymerase chain reaction. The 2‐deoxy‐d‐glucose‐6‐phosphate (2‐DG6P) level was detected utilizing 2‐deoxy‐d‐glucose (2‐DG) uptake measurement kit. Western blot was adopted to measure the protein expressions of insulin‐like growth factor‐1 receptor (IGF‐1R), G6Pase, PEPCK, and phosphatidylinositol 3‐kinase (PI3K)/Akt pathway‐related genes. StarBase was used to predict the targeting relationship between lncRNA XIST or IGF‐1R with miR‐182‐5p, the results of which were verified by dual‐luciferase reporter, RNA pull‐down, and RNA immunoprecipitation assays. Rescue experiments were conducted to investigate the effect of miR‐182‐5p on IR cells. Next, low‐expressed lncRNA XIST and high‐expressed miR‐182‐5p were observed in IR cells.

**Results:**

Upregulation of lncRNA XIST increased IGF‐1R and 2‐DG6P levels, decreased G6Pase and PEPCK expressions, and promoted PI3K/Akt pathway activation in IR cells. LncRNA XIST sponged miR‐182‐5p which targeted IGF‐1R. MiR‐182‐5p mimic reversed the above effects of lncRNA XIST overexpression on IR cells.

**Conclusions:**

In conclusion, lncRNA XIST/miR‐182‐5p axis alleviates hepatic IR in vitro via IGF‐1R/PI3K/Akt signaling pathway, which could be the promising therapeutic target.

## 1 INTRODUCTION

Insulin resistance (IR) refers to a decrease in the responsiveness of cells or tissues to normal doses of insulin. IR usually precedes obvious pathological conditions, such as type II diabetes (T2D) or metabolic syndrome, and is associated with conditions incorporating overweight or obesity.^1^, ^2^ In recent years, the incidence of IR‐related diseases such as T2D has been in an uptrend due to lifestyle changes, including reduced physical exercise and increased food intake.^3^ Therefore, it is necessary to correctly understand the pathogenesis of IR and find treatment options.

Long noncoding RNAs (lncRNAs) are involved in regulating many biological processes, including genetic imprinting, transcriptional regulation, and posttranscriptional regulation.^4^ Numerous studies indicated that lncRNAs own crucial functions in IR. For instance, lncRNA H19 has a promoting effect on skeletal muscle insulin sensitivity,^5^ and lncRNA TUG1 downregulation decreases insulin secretion in mouse pancreatic β cells.^6^ LncRNA X‐inactive specific transcript (XIST) gene resides in the X inactivation center (Xic) and encodes RNA longer than 200 bp rather than protein.^7^ The existing studies have elucidated that XIST plays a vital role in a variety of tumors. Specifically, lncRNA XIST is highly expressed in glioblastoma, knockdown of which induced by miR‐152 inhibits the growth, invasion, and migration of glioma cells and induces apoptosis.^8^ In addition, lncRNA XIST has an impact on poor glycemic control and IR,^9^ and the expression of XIST is negatively correlated with IR,^10^ so we focused on exploring the mechanism of XIST in IR.

Insulin‐like growth factors (IGFs) are widely distributed in various tissues of the body, among which IGF‐1 is an important member.^11^ IGF‐1R is the receptor of IGF‐1, which is expressed in fat, liver, and bone.^12^ A study has found that downregulated IGF‐1R leads to enhanced insulin‐mediated glucose uptake.^13^ In addition, IGF‐1 intensifies the insulin sensitivity of liver and muscle, and improves T2D by binding with IGF‐1R located on the cell membrane that activates downstream PI3K/Akt pathway, an important insulin signaling pathway.^14^ The above literatures suggested that changes in IGF‐1R expression may be an important link in the progression of IR.

Therefore, we hypothesized that XIST may suppress IR through the regulation of IGF‐1R, and this study aimed to examine specifically how this regulatory relationship is achieved. The goal of our study is to provide a new direction and possibility for the treatment of IR‐related diseases such as T2D and metabolic syndrome.

## 2 MATERIALS AND METHODS

### 2.1 Establishment of IR cell model

LX‐2 cells (SCC064; Sigma‐Aldrich) were cultured in minimum essential medium (12360038; Gibco) with 10% fetal bovine serum (FBS, 16140071; Gibco) at 37°C with 5% CO_2_. The IR cell model was established as follows^15^: LX‐2 cells were processed by serum‐starving for 12 h (h), followed by treatment with 18 mM glucosamine (PHR1199; Sigma‐Aldrich) for 18 h in the serum‐free medium. Finally, cells were stimulated with 100 nM insulin (I2643; Sigma‐Aldrich) for 30 min (min).^16^

### 2.2 Transfection

LncRNA XIST was amplified and inserted into the pCMV6 vector (PS100001; OriGene) to obtain pCMV6‐XIST vector. XIST short hairpin RNA (shXIST, C02001, cagaagaatggtacaaatccaag) and shRNA negative control (shNC, C03002) were purchased from GenePharma. Next, 2.5 μg of pCMV6‐XIST, pCMV6 empty vector, shXIST, and shNC, 100 pmol of miR‐182‐5p mimic (M, miR10000259‐1‐5; Ribobio) and mimic control (MC, miR1N0000003‐1‐5; Ribobio), and 5 μL of Lipo6000 transfection reagent (C0526; Beyotime) were, respectively, diluted in 125 μL of Opti‐MEM (31985062; Thermo Fisher Scientific), which were then mixed together and added to six‐well plate (3 × 10^5^ cells/well) for 24‐h transfection. Finally, transfected cells could be analyzed by quantitative reverse transcription polymerase chain reaction (qRT‐PCR).

### 2.3 Cell grouping

LX‐2 cells were divided into two parts. Five groups were constructed in the first part: blank group (normally cultured cells), IR group (IR model cells), empty vector control (EVC) group (IR model cells transfected with EVC and shNC), XIST group (IR model cells transfected with XIST overexpression plasmid), and shXIST group (shXIST‐transfected IR model cells). Six groups were established in the second part, namely, blank group, IR group, EVC + MC group (IR model cells transfected with EVC plus miR‐182‐5p MC), EVC + M group (IR model cells transfected with EVC plus miR‐182‐5p mimic), XIST + MC group (IR model cells transfected with XIST overexpression plasmid plus miR‐182‐5p MC), and XIST + M group (IR model cells transfected with XIST overexpression plasmid plus miR‐182‐5p mimic).

### 2.4 QRT‐PCR

Total RNA from LX‐2 cells was extracted using Trizol reagent (15596026; Invitrogen). qRT‐PCR was performed using Hifair® Ⅲ One Step RT‐qPCR SYBR Green Kit (11143ES50; Yeasen) on an ABI7500 Real‐Time PCR instrument (Applied Biosystems) in accordance with the manufacturer' instructions. The assays were repeated in triplicate, and the gene expressions were calculated by the $2^{‐∆∆C_{t}}$ method.^17^ The primer sequences were presented in Table 1. Glyceraldehyde‐3‐phosphate dehydrogenase (GAPDH) was served as the normalization control of lncRNA XIST, glucose‐6‐phosphatase (G6Pase), and phosphoenolpyruvate carboxykinase (PEPCK). U6 worked as the normalization control of miR‐182‐5p.

**Table 1:** Primers for quantitative reverse transcription polymerase chain reaction.

Gene names	Forward primer (5′–3′)	Reverse primer (5′–3′)
LncRNA XIST	CCATTGAAGATACCACGCTGC	GGTTGTTGCCCAGGGTAGTG
miR‐182‐5p	ATCACTTTTGGCAATGGTAGAACT	TATGGTTTTGACGACTGTGTGAT
G6Pase	CCTACAGATTTCGGTGCTTG	GATGCTGTGGATGTGGCTGA
PEPCK	CCCTGGGAGATGGTGACTTT	CGCTGCCGAAGGAGATGA
GAPDH	CTCAACTACATGGTCTACATGTTCCA	CTTCCCATTCTCAGCCTTGACT
U6	CTCGCTTCGGCAGCACA	AACGCTTCACGAATTTGCGT

Abbreviations: GAPDH, glyceraldehyde‐3‐phosphate dehydrogenase; PEPCK, phosphoenolpyruvate carboxykinase; XIST, X‐inactive specific transcript.

### 2.5 Target gene prediction and verification

The binding sites between lncRNA XIST/IGF‐1R and miR‐182‐5p were predicted by starBase (http://starbase.sysu.edu.cn/) and verified by dual‐luciferase reporter assay. Briefly, the wild‐type lncRNA XIST or IGF‐1R was inserted into the pmirGLO vector (XIST/IGF‐1R‐WT, E1330; Promega). The mutated lncRNA XIST or IGF‐1R was obtained by point mutation and inserted into the pmirGLO vector (XIST/IGF‐1R‐MUT). The gene sequences were listed below: XIST‐WT, 5′‐GAGUAAAAUUCUAAUUGCCAAU‐3′, XIST‐MUT, 5′‐GAGUAAAAUACUCAUCGCGAAU‐3′; IGF‐1R‐WT, 5′‐UUAACGCUGCCUAAUUUUGCCAAA‐3′, IGF‐1R‐MUT, 5′‐UUAACGCUGCCAAAUUUCGCAAAA‐3′. Next, 100 pmol of miR‐182‐5p mimic or MC and 50 ng of XIST/IGF‐1R‐WT or XIST/IGF‐1R‐MUT were co‐transfected into LX‐2 cells. Forty‐eight hours later, the dual‐luciferase reporter assay was completed followed the protocol for the Dual‐Luciferase Reporter Assay System (E1910; Promega), and the ratio of firefly to *Renilla* luciferase activities was calculated to determine relative luciferase activity.

### 2.6 RNA‐biotin pull‐down assay

According to the previous study,^10^ LX‐2 cells were treated with miR‐182‐5p‐WT/MUT that labeled with the Biotin RNA Labeling Mix (11685597910; Roche). After 48 h, cells were lysed by RIPA buffer (R0020; Solarbio) for 10 min and then DNase I (RNase‐free, 69182‐3; Millipore) was used to treat cell lysates, followed by the incubation with streptavidin‐coated magnetic beads (08014; Sigma‐Aldrich) at 4°C for 3 h. Thereafter, the extracted RNA from complexes was used for qRT‐PCR.

### 2.7 RNA immunoprecipitation (RIP)

The procedures of RIP were conducted with reference to the previous study.^10^ LX‐2 cells were lysed for 30 min at 4°C. Next, cell lysates underwent incubation with magnetic beads conjugated with Argonaute 2 (Ago2) antibody (ab186733; Abcam) or anti‐IgG (ab6715; Abcam) as negative control at 4°C overnight. Later, the samples were digested with DNase I and Proteinase K, followed by the isolation of immunoprecipitated RNA. Finally, RNA enrichment was determined by qRT‐PCR.

### 2.8 Western blot

Western blot assay was proceeded based on the previous study.^18^ Simply put, total protein of LX‐2 cells was extracted with RIPA lysis buffer (E‐BC‐R327; Elabscience). After the protein concentrations were measured by BCA protein assay, 30 μg of the protein was separated by 12% sodium dodecyl sulfate‐polyacrylamide gel electrophoresis (SDS‐PAGE) gels, and transferred onto polyvinylidene fluoride membranes (3010040001; Roche). Then, the membranes were blocked, followed by the incubation with primary antibodies at 4°C overnight and a secondary antibody at 37°C for 1 h. The protein band was exposed with ECL buffer (R30199; Pierce), and the intensity analysis was achieved with iBright FL1500 Imaging System (A44115; Invitrogen). The primary antibodies used were those against G6Pase (1:1000; Rabbit; ab93857; abcam, 40 kDa), PEPCK (1:1000; Rabbit; No. ABIN2855891; antibodies‐online, 71 kDa), p‐PI3K (1:1000; Rabbit; #029801; Alamo Laboratories Inc, 85 kDa), PI3K (1:1000; Rabbit; #4249; Cell Signaling Technology (CST), 110 kDa), p‐Akt (1:2000; Rabbit; #4060; CST, 60 kDa), Akt (1:1000; Rabbit; #4685; CST, 60 kDa), insulin‐like growth factor‐1 receptor (IGF‐1R, 1:1000; Rabbit; ab182408; abcam, 156 kDa) and GAPDH (1:10000; Rabbit; ab181602; abcam, 36 kDa). The secondary antibody was goat anti‐rabbit antibody (1:2000, #14708; CST). GAPDH was served as a loading control.

### 2.9 Measurement of cell glucose uptake

The concentration of 2‐deoxy‐d‐glucose‐6‐phosphate (2‐DG6P) in LX‐2 cells was measured utilizing 2‐deoxy‐d‐glucose (2‐DG) uptake measurement kit (CRS‐OKP‐PMG‐K01E; Cosmo Bio) to detect the cell glucose uptake following the manufacturer's directions. LX‐2 cells were seeded in a 96‐well plate at a density of 1500 cells/well, differentiated, and then maintained for 4 days before use. The cells were starved for glucose by preincubating with 100 µL of Krebs‐Ringer‐Phosphate‐HEPES buffer (MG6617; MesGen Biotech) containing 2% bovine serum albumin (BSA) for 40 min, and then stimulated or not with 1 μM insulin for 20 min to activate the glucose transporter. Next, cells were treated with 10 µL of 10 mM 2‐DG and incubated for 20 min. According to the manufacturer's specification, appropriate reagent was applied to induce nicotinamide adenine dinucleotide phosphate generation which was the detection index of 2‐DG6P content. The absorbance values were measured at 412 nm utilizing a microplate reader (E0228; Beyotime).

### 2.10 Statistical analysis

The measurement data were expressed as mean ± standard deviation (SD) and analyzed by SPSS 17 statistical software (IBM). One‐way analysis of variance was introduced for multiple group comparisons, and Bonferroni test was applied for further analysis. The *p* < .05 was considered to be statistical significance.

## 3 RESULTS

In this study, we hypothesized that lncRNA XIST may act through regulation of IGF‐1R to suppress IR, and this study aimed to explore how this regulatory relationship is achieved. First, LX‐2 cells were applied to establish an IR model in vitro. Then, we performed a series of experiments, such as qRT‐PCR, 2‐DG uptake measurement test, western blot, and rescue experiments, to analyze the role of lncRNA XIST on IR model cells and its underlying mechanism. Finally, this work demonstrated that lncRNA XIST alleviates hepatic IR via regulating the miR‐182‐5p/IGF‐1R axis, which could be the promising therapeutic target of IR‐related diseases such as T2D and metabolic syndrome.

### 3.1 LncRNA XIST‐regulated IGF‐1R, 2‐DG6P, G6Pase, and PEPCK expressions and PI3K/Akt pathway in IR cells

**Figure 1:**
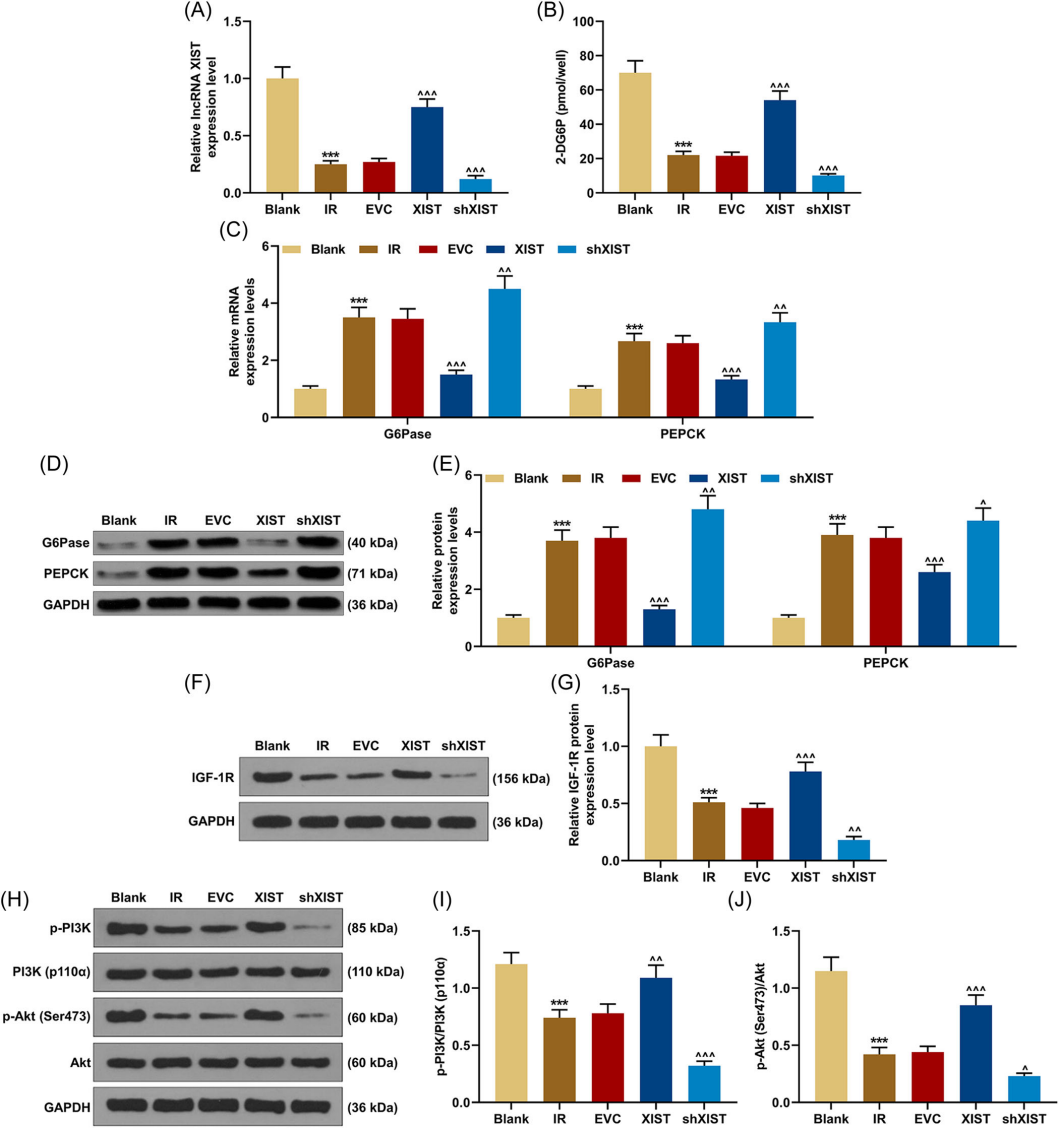
Effects of lncRNA XIST on IGF‐1R, 2‐DG6P, G6Pase, and PEPCK expressions and PI3K/Akt pathway in IR cells. (A) LncRNA XIST expression was detected by qRT‐PCR, and GAPDH was served as the internal reference. (B) The concentration of 2‐DG6P in LX‐2 cells was detected by 2‐DG uptake measurement kit. (C) The mRNA expression levels of G6Pase and PEPCK were determined by qRT‐PCR, and GAPDH was served as the internal reference. (D–E) Western blot was used to detect the G6Pase and PEPCK protein expressions, and GAPDH was served as the internal reference. (F and G) The protein expression of IGF‐1R was also assessed by western blot, and GAPDH was served as the internal reference. (H–J) Western blot was performed again to measure the protein expressions of PI3K, p‐PI3K, Akt, and p‐Akt, and GAPDH was served as the internal reference. All experiments were repeated three times to average. The data were presented as the mean ± standard deviation (SD) of three independent experiments; ***p < .001 versus Blank; ^*p* < .05; ^^*p* < .01; ^^^*p* < .001 versus EVC. 2‐DG6P, 2‐deoxy‐d‐glucose‐6‐phosphate; EVC, empty vector control; G6Pase, glucose‐6‐phosphatase; GAPDH, glyceraldehyde‐3‐phosphate dehydrogenase; IGF‐1R, insulin‐like growth factor‐1 receptor; IR, insulin resistance; PEPCK, phosphoenolpyruvate carboxykinase; PI3K, phosphatidylinositol 3‐kinase; qRT‐PCR, quantitative reverse transcription polymerase chain reaction; shXIST, XIST short hairpin RNA; XIST, X‐inactive specific transcript.

When comparing lncRNA XIST expression level in different groups, it could be observed that lncRNA XIST expression was lower in IR group than in blank group (*p* < .001, Figure 1A), but was higher in XIST group than in the EVC group (*p* < .001, Figure 1A). Moreover, compared to the EVC group, the lower lncRNA XIST expression was observed in the shXIST group (*p* < .001, Figure 1A). As shown in Figure 1B, the content of 2‐DG6P was reduced in IR group, which was also higher in XIST group but was lower in shXIST group as compared to that in EVC group (*p* < .001, Figure 1B). Additionally, IR group presented higher expressions of G6Pase and PEPCK and lower IGF‐1F expression in contrast to blank group (*p* < .001, Figure 1C–G). From the comparison results, it could be concluded that XIST overexpression brought about downregulated G6Pase a–Gnd PEPCK yet upregulated IGF‐1R (*p* < .001, Figure 1C–G), while silencing of XIST oppositely regulated these expressions (*p* < .05, Figure 1C–G). Furthermore, the ratios of p‐PI3K/PI3K and p‐Akt/Akt in IR group were lower than those in blank group (*p* < .001, Figure 1H–J), AND overexpressed or silenced XIST led to the induction or reduction of p‐PI3K/PI3K and p‐Akt/Akt, respectively (*p* < .05, Figure 1H–J). These data indicated that lncRNA XIST overexpression weakened the impact of IR, facilitated the activation of PI3K/Akt pathway, augmented the levels of 2‐DG6P and IGF‐1R, and inhibited the expressions of G6Pase and PEPCK in cells, revealing that XIST overexpression can promote the hypoglycemic and insulin‐sensitizing capabilities of IR cells.

### 3.2 LncRNA XIST directly sponged miR‐182‐5p which was targeted by IGF‐1R

**Figure 2:**
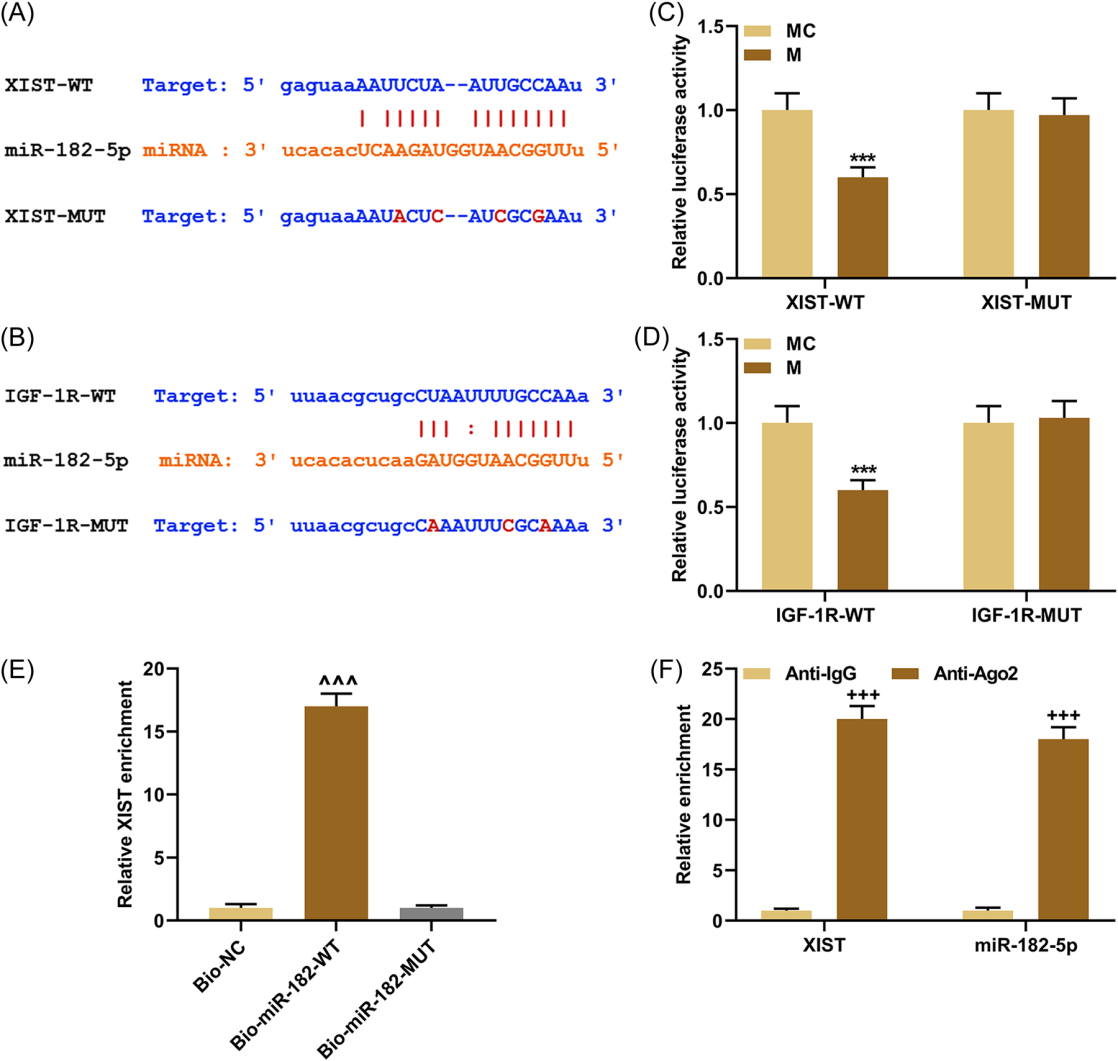
The target relationship between lncRNA XIST/IGF‐1R and miR‐182‐5p. (A and B) StarBase (http://starbase.sysu.edu.cn/) was used to predict the targeted binding sites between lncRNA XIST/IGF‐1R and miR‐182‐5p. (C and D) Relative luciferase activity of wild‐type lncRNA XIST/IGF‐1R (XIST/IGF‐1R‐WT) and mutant lncRNA XIST/IGF‐1R (XIST/IGF‐1R‐MUT) groups. (E) The enrichment of XIST in the Bio‐NC, Bio‐miR‐182‐WT, and Bio‐miR‐182‐MUT groups was determined by RNA‐biotin pull‐down assay. (F) The enrichment of XIST and miR‐182‐5p in the Anti‐IgG and Anti‐Ago2 groups was detected by RNA immunoprecipitation assay. All experiments were repeated three times to average. The data were presented as the mean ± standard deviation (SD) of three independent experiments; ***p < .001 versus MC; ^^^*p* < .001 versus Bio‐miR‐182‐MUT; ^+++^ *p* < .001 versus Anti‐IgG. Ago2, Argonaute 2; Bio, biotin; IGF‐1R, insulin‐like growth factor‐1 receptor; MC, mimic control; XIST, X‐inactive specific transcript.

According to starBase, there were targeted binding sites between lncRNA XIST/IGF‐1R and miR‐182‐5p (Figure 2A,B), indicating the potentiality that lncRNA XIST can target miR‐182‐5p and miR‐182‐5p may target IGF‐1R. After co‐transfection of XIST/IGF‐1R‐WT with miR‐182‐5p mimic into cells, the luciferase activity was obviously decreased compared to co‐transfection of that with miR‐182‐5p MC (*p* < .001, Figure 2C,D). Besides, the luciferase activity remained unchanged after co‐transfection of XIST/IGF‐1R‐MUT with miR‐182‐5p mimic or MC into cells (Figure 2C,D). Thereafter, the results of RNA pull‐down and RIP experiments displayed that XIST enrichment was remarkably upregulated in the Bio‐miR‐182‐WT group (*p* < .001, Figure 2E) AND the Anti‐Ago2 group (*p* < .001, Figure 2F), AND the miR‐182‐5p enrichment was also increased in the Anti‐Ago2 group (*p* < .001, Figure 2F). The above discoveries proved the target relationship between XIST/IGF‐1R and miR‐182‐5p.

### 3.3 The effects of lncRNA XIST overexpression on the insulin metabolism‐related proteins and PI3K/Akt pathway were partially reversed by miR‐182‐5p mimic

**Figure 3:**
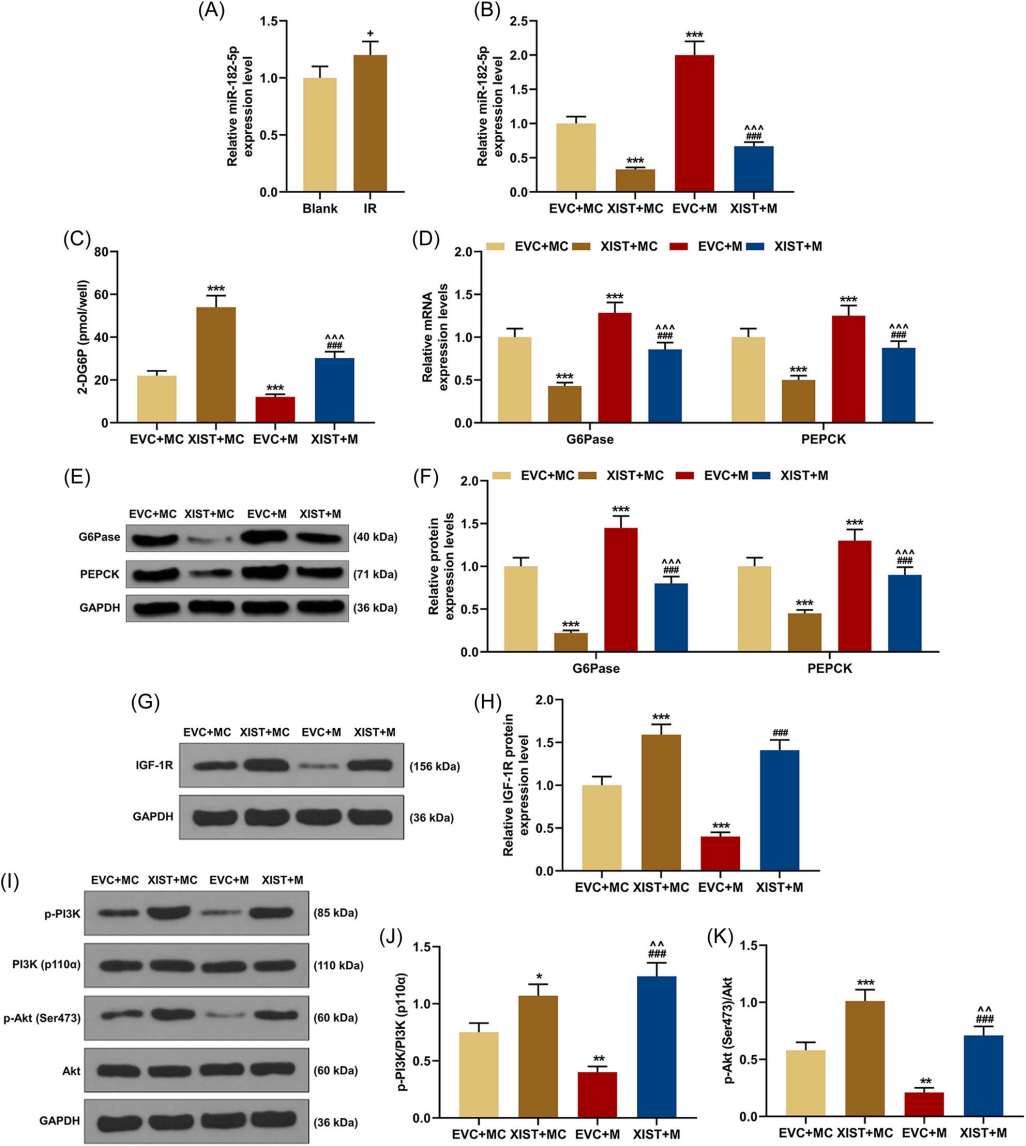
Effects of miR‐182‐5p on IGF‐1R, 2‐DG6P, G6Pase and PEPCK expressions and PI3K/Akt pathway in IR cells. (A) MiR‐182‐5p expression was detected by qRT‐PCR, with U6 as the internal reference. (B) The miR‐182‐5p expression level in EVC + MC, XIST + MC, EVC + M, and XIST + M groups were detected by qRT‐PCR, with U6 serving as the internal reference. (C) The 2‐DG6P content was detected by 2‐DG uptake measurement kit. (D) After transfection, the mRNA expressions of G6Pase and PEPCK were determined by qRT‐PCR, with GAPDH serving as the internal reference. (E–H) The protein expressions of G6Pase, PEPCK, and IGF‐1R were measured by western blot, with GAPDH serving as the internal reference. (I–K) The PI3K/Akt pathway‐related proteins were also detected by western blot, with GAPDH serving as the internal reference. All experiments were repeated three times to average. The data were presented as the mean ± standard deviation (SD) of three independent experiments; ^+^ *p* < .05 versus Blank; *p < .05; ***p < .001 versus EVC + MC; ^^*p* < .01; ^^^*p* < .001 versus XIST + MC; ^###^ *p* < .001 versus EVC + M. 2‐DG6P, 2‐deoxy‐d‐glucose‐6‐phosphate; EVC, empty vector control; GAPDH, glyceraldehyde‐3‐phosphate dehydrogenase; G6Pase, glucose‐6‐phosphatase; IGF‐1R, insulin‐like growth factor‐1 receptor; IR, insulin resistance; M, miR‐182‐5p mimic; MC, mimic control; PEPCK, phosphoenolpyruvate carboxykinase; PI3K, phosphatidylinositol 3‐kinase; qRT‐PCR, quantitative reverse transcription polymerase chain reaction; XIST, X‐inactive specific transcript.

It could be easily discovered that miR‐182‐5p expression was upregulated in IR cells (*p* < .05, Figure 3A) and was further elevated by miR‐182‐5p mimic (*p* < .001, Figure 3B), while overexpressed lncRNA XIST had the ability to down‐regulate miR‐182‐5p expression in IR cells (*p* < .001, Figure 3B). Moreover, miR‐182‐5p mimic reversed the effect of XIST overexpression, which diminished 2‐DG6P concentration, and induced the expressions of G6Pase and PEPCK in IR cells (*p* < .001, Figure 3C–F). Additionally, miR‐182‐5p mimic suppressed IGF‐1R expression and the ratios of p‐PI3K/PI3K and p‐Akt/Akt (*p* < .001, Figure 3G–K), AND partially neutralized the effect of XIST overexpression on p‐Akt/Akt. However, its reversal effects on overexpressed XIST‐induced high levels of IGF‐1R expression and p‐PI3K/PI3K were not significant (Figure 3G–K).

## 4 DISCUSSION

Ample research have been carried out to unravel the correlation between lncRNA XIST and diabetes. Generally speaking, lncRNA XIST has a high expression in coronary artery disease patients with T2D,^19^ and silencing of lncRNA XIST protects against renal interstitial fibrosis in diabetic nephropathy^20^ and alleviates inflammation in diabetic nephropathy.^21^ The current study found that lncRNA XIST had a low expression in IR model cells. Also, overexpressed lncRNA XIST increased 2‐DG6P content, promoted IGF‐1R expression and inhibited insulin metabolism‐related protein expressions (PEPCK and G6Pase), while silencing of XIST exerted an entirely opposite role, implying that overexpressed lncRNA XIST had an inhibitory effect on hepatic IR.

It has been reported that lncRNA acts as a “molecular sponge” and directly or indirectly competitively binds to microRNA (miRNA), which will eventually lead to the weakening of the interaction between miRNA and messenger RNA.^22^ According to the prediction of bioinformatics, lncRNA XIST has the potential to interact with miR‐182‐5p, and our study also proved that XIST can directly target miR‐182‐5p. Combined with the previous studies that miR‐122‐5p affects IR in hepatocytes by targeting IGF‐1R and miR‐182‐5p inhibits IGF‐1R expression in hepatocellular carcinoma,^15^, ^23^ we conjectured that miR‐182‐5p may regulate hepatic IR by binding to IGF‐1R, which was then confirmed by our experimental results. Thus, miR‐182‐5p was a common target of XIST and IGF‐1R in hepatic IR.

miRNAs are a kind of short endogenous noncoding RNAs which could take part in the regulation of the intracellular signaling pathways involved in diseases.^24^ MiRNAs are not only involved in regulating many important physiological processes such as cell differentiation, proliferation, and programmed death, but also in modulating insulin production, secretion and effect, and glucose homeostasis.^25^ It has been demonstrated that miR‐26a regulates insulin sensitivity and metabolism of glucose^26^ and miR‐199a‐5p inhibits insulin sensitivity in hepatocytes via suppressing ATG14‐mediated autophagy.^27^ Moreover, Weale et al. pointed out that the expression of miR‐182‐5p is increased in patients with prediabetes or T2D and it may potentially predict T2D.^28^ In this study, the expression of miR‐182‐5p was increased in IR cells, and miR‐182‐5p mimic partially reversed the effect of lncRNA XIST overexpression, lessening the levels of IGF‐1R and 2‐DG6P and augmenting the expressions of insulin metabolism‐related proteins. On the above basis, we focused on the downstream pathway that regulated by IGF‐1R.

As previously mentioned, PI3K/Akt signaling pathway is the pathway activated by IGF‐1R, which plays a significant role in blood glucose regulation, glucose transporter activation, and initiation of glucose storage or decomposition in cells.^29^ Heterozygotes with knockouts of the regulatory subunits p85, β55, and 50α of PI3K have reinforced sensitivity to insulin, leading to the development of hypoglycemia.^30^ Knockout of Akt2 could cause IR and diabetes in the liver and skeletal muscle.^31^ Cheng et al. proved that lncRNA XIST mediates glucose metabolism via the PI3K/Akt pathway in glioma.^10^ In addition, it was found that Akt signaling would not change significantly with the change of hepatocyte insulin sensitivity.^32^, ^33^ Similarly, our study corroborated that lncRNA XIST overexpression promoted the expression of IGF‐1R, and then activated the PI3K/AKT signaling pathway, but miR‐182‐5p mimic reversed its effects, manifesting that lncRNA XIST/miR‐182‐5p axis could restore the function of IR cells in regulating blood glucose via upregulating IGF‐1R level to activate PI3K/Akt signaling pathway.

Abnormal hepatic glucose metabolism is the main pathological basis of metabolic diseases such as diabetes and obesity, with hepatic IR as the main pathogenesis of such diseases. The most obvious pathophysiological characteristics of hepatic IR refer to that disorders of gluconeogenesis and glycogen catabolism lead to increased hepatic glucose output, of which the role of gluconeogenesis is particularly significant.^30^ 2‐DG, a glucose isomer, can inhibit virus infection, yeast fermentation, pathogenic bacteria, and tumor cell growth.^34^ 2‐DG can be absorbed by cells and phosphorylated by hexokinase II to 2‐DG6P. However, 2‐DG6P, as a substrate for glycogen synthesis, cannot continue to participate in glucose metabolism, but can inhibit the function of hexokinase and induce cell death as a glycolysis inhibitor.^35^ In our study, lncRNA XIST overexpression upregulated 2‐DG6P content in IR cells, which indicated a strengthening glucose uptake capacity, and the decreased 2‐DG6P level induced by miR‐182‐5p mimic represented the weakening glucose uptake capacity.

Insulin regulates protein expressions and activity to maintain blood glucose within normal limits. PEPCK and G6Pase are important enzymes in the process of hepatic gluconeogenesis and hepatic glucose export, and the rate‐limiting enzymes in the final reaction of gluconeogenesis and glycogen decomposition,^36^ whose upregulation can promote glycolysis to produce glucose and lead to glycogen storage.^37^ From the confirmation in our study, the induced expressions of PEPCK and G6Pase in IR cells were decreased by lncRNA XIST overexpression but miR‐182‐5p mimic could reverse the effects of lncRNA XIST overexpression, which meant that the lncRNA XIST‐miR‐182‐5p interaction network inhibited glycolysis and glucose production.

At present, it has been found that IR is closely related to liver fibrosis, and anti‐IR therapy can alleviate liver fibrosis.^38^ The characteristic of liver fibrosis is an increase in extracellular matrix (ECM) synthesis and a decrease in degradation, resulting in a large amount of fibrous tissue settling in the liver.^39^ During the development of liver fibrosis, activated hepatic stellate cells are the key cells that produce ECM.^39^ Our study used LX‐2 cells (hepatic stellate cells) to construct an IR cell model and found that overexpression of lncRNA XIST could inhibit IR in vitro, so lncRNA XIST may also have a reducing effect on liver fibrosis, but this hypothesis still needs further validation.

To sum up, lncRNA XIST overexpression could improve insulin metabolism and ameliorate IR of hepatocytes through inhibiting miR‐182‐5p to activate IGF‐1R/PI3K/Akt signaling pathway, which could be utilized as a promising biomarker in clinical treatment of IR‐related diseases. In the future, we will conduct rescue experiments about the interaction between lncRNA XIST and IGF‐1R in IR as well as animal experiments to further refine our findings.

## AUTHOR CONTRIBUTIONS

**Guoqing Zhong and Qingping Yang**: Conceptualization (equal); writing—original draft (lead); writing—review and editing (lead). **Yihua Wang, Yuan Liang, Xiaojing Wang, and Dongli Zhao**: Formal analysis (equal); data curation‐(equal); methodology (equal); writing—review and editing (supporting); writing—original draft (supporting).

## CONFLICT OF INTEREST STATEMENT

The authors declare no conflict of interest.

## Data Availability

The analyzed data sets generated during the study are available from the corresponding author on reasonable request.
